# A “two-hit” (chemo)therapy to improve checkpoint inhibition in cancer

**DOI:** 10.18632/oncoscience.533

**Published:** 2021-05-07

**Authors:** Paolo Falvo, Stefania Orecchioni, Alessandro Raveane, Giulia Mitola, Francesco Bertolini

**Affiliations:** ^1^ Laboratory of Hematology-Oncology, European Institute of Oncology IRCCS, Milan 20141, Italy; ^*^ Contributed equally to this work

**Keywords:** anti-PD-1, immunotherapy, breast cancer, lymphoma, chemotherapy, T-cell therapy

In the last decade, anti-PD-1 and anti-PD-L1 checkpoint inhibitors (CIs) have demonstrated to be clinically active as a single agent in several types of solid tumors and hematological malignancies. However, the clinical benefit is observed only in a fraction of patients and, in the majority of cases, only for a limited period of time [1]. These observations, along with the evidence of multilayered complexity in cancer cells, cancer microenvironment and anti-cancer immunity, have promoted preclinical and clinical studies with combinatorial drugs aimed at improving CI activity by re-shaping the immune system before and during CI administration [2].

Chemotherapeutics are known to have profound effects over several myeloid, innate, helper and effector immune cell subsets [3]. As CI clinical activity is dependent upon a coordinated activity between tumor antigen processing by some myeloid antigen-presenting cells (APCs), and cancer cell targeting by helper and effector T cells [4], we investigated *in vivo* in immunocompetent murine models of triple negative breast cancer (TNBC) and B-cell lymphoma the effects of different types and dosages of chemotherapeutics over the immune cell orchestra.

Two main findings emerged: first, we observed that vinca alcaloids (V), at low dosages, can generate and activate new APCs. Secondly, we found that intermitted, medium dosage cyclophosphamide (140 mg/Kg every 6 days, C140) can generate new CD3+CD4+ and CD3+CD8+T cell clones. In our models, a “two-hit” approach (V plus C140) significantly improved CI preclinical activity against local and disseminated neoplastic growth [5-6].

Our models included mice with neoplastic lesions showing an immune infiltrate predominantly including lymphoid cells and others with and an immune infiltrate with high proportions of myeloid cells. In both cases, antibody-mediated depletion of CD3+CD4+ or of CD3+CD8+ T cells abrogated the preclinical efficacy of the V plus C140 plus CI combinatorial therapy. These data suggest that anti-cancer activity was largely related to these immune T cells [6].

**Figure 1:**
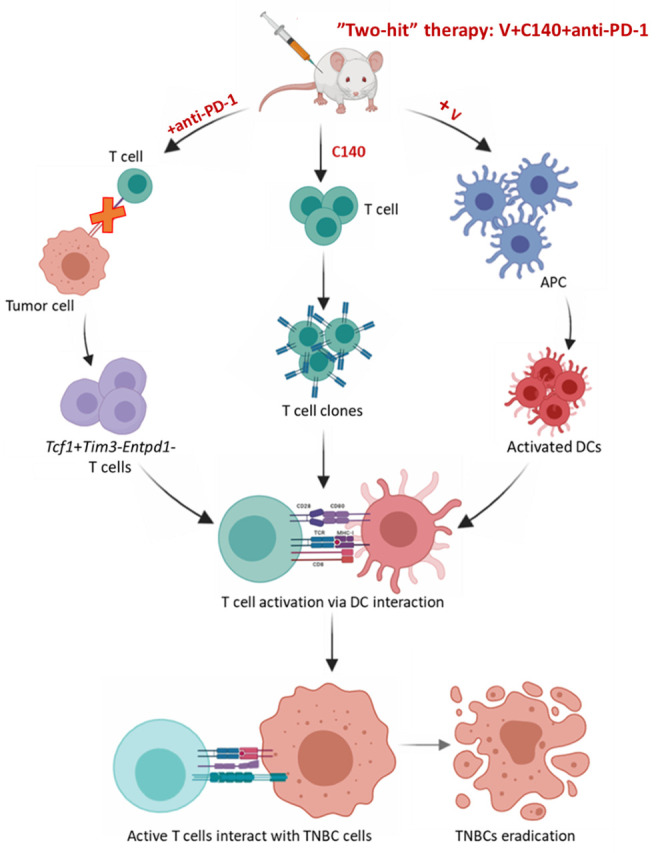
**Figure 1: Graphical abstract of the preclinical effects of the “two hit” therapy (V plus C140 and anti-PD1) over APC, T and cancer cells in TNBC models.** “The image was generated using BioRender software (https://biorender.com/)”

Single-cell transcriptome analysis of >50,000 intratumoural immune cells, after V plus C140 and CI combinatorial therapy, showed a gene signature suggestive of a change resulting from exposure to a mitogen, ligand, or an antigen for which it is specific, as well as APC-to-T-cell adhesion [6]. This transcriptional program also significantly increased the number of intratumoural *Tcf1*+ stem-like CD8+ T-cells [7] and altered the balance between terminally and progenitor exhausted T-cells, favoring the latter [6]. The proposed mechanism of V plus C140 and CI combinatorial therapy is showed in (Figure 1)

As V and C140 dosages found to significantly improve CI preclinical activity are suitable for a combinatorial use in cancer patients, clinical trials in TNBC and B-cell lymphoma are now planned to confirm the efficacy of this “two-hit” plus CI therapy. It will be of interest to investigate in enrolled patients a number of emerging candidate biomarkers of immune cell activation, including: a) APC activation patterns; b) the generation of new *TCF1*+ stem-like CD8+ T-cells; c) *CXCL13* and *CCR5* overexpressing, neo-antigen reactive CD8+ T cells [8]; d) newly generated IFN-γ–expressing resident memory T cells [9]; e) pre-treatment values of senescent CD28-CD57+KLGR1+CD8+ T cells [10]; and f) post-treatment values of proliferating PD-1+CD8+ T cells [11]. The goal of such a therapy, in fact, is the generation of a long-lasting anti-tumor immunity in treated cancer patients.
